# Axillary surgery in patients with sentinel node macrometastases: secondary results of the randomized INSEMA trial

**DOI:** 10.1038/s41523-026-00902-7

**Published:** 2026-01-27

**Authors:** Toralf Reimer, Angrit Stachs, Kristina Veselinovic, Thorsten Kuehn, Joerg Heil, Silke Polata, Frederik Marme, Elisabeth Trapp, Guido Hildebrandt, David Krug, Beyhan Ataseven, Roland Reitsamer, Sylvia Ruth, Carsten Denkert, Inga Bekes, Nicole Stahl, Marc Thill, Hans-Joachim Strittmatter, Thomas Mueller, Michael Golatta, Dirk-Michael Zahm, Johannes Holtschmidt, Michael Knauer, Valentina Nekljudova, Sibylle Loibl, Bernd Gerber

**Affiliations:** 1 Department of Obstetrics and Gynecology, University of Rostock, Rostock, Germany; 2 University Hospital Ulm, Ulm, Germany; 3 Hospital Esslingen, Esslingen, Germany; 4 University Hospital Heidelberg, Heidelberg, Germany; 5 Breast Unit, Sankt Elisabeth Hospital, Heidelberg, Germany; 6 Evangelical Forest Hospital Spandau, Berlin, Germany; 7 Department of Obstetrics and Gynecology, Faculty of Medicine Mannheim, University Heidelberg, Mannheim, Germany; 8 Department of Obstetrics and Gynecology, University Hospital, Graz, Austria; 9 Department of Radiation Oncology, University Hospital Rostock, Rostock, Germany; 10 Department of Radiotherapy and Radiation Oncology, University Hospital Hamburg-Eppendorf (UKE), Hamburg, Germany; 11 Department of Radiation Oncology, University Medical Center Schleswig-Holstein, Kiel, Germany; 12 Department of Gynecology and Obstetrics, Medical School and University Medical Center OWL, Klinikum Lippe, Bielefeld University, Detmold, Germany; 13 Salzburg Regional Hospital, Salzburg, Austria; 14 Johanniter-Hospital Genthin-Stendal, Stendal, Germany; 15 Institute of Pathology, Philipps-University Marburg and University Hospital Marburg (UKGM), Marburg, Germany; 16 Breast Center Kantonsspital St. Gallen, St. Gallen, Switzerland; 17 Breast Unit, Helios Clinic, Schwerin, Germany; 18 Department of Gynecology and Gynecologic Oncology, Agaplesion Markus Hospital, Frankfurt am Main, Germany; 19 Rems-Murr-Hospital, Winnenden, Germany; 20 Hanau City Hospital, Hanau, Germany; 21 SRH Wald-Klinikum, Gera, Germany; 22 German Breast Group, Neu-Isenburg, Germany; 23 Tumor and Breast Center Eastern Switzerland, St. Gallen, Switzerland

**Keywords:** Cancer, Diseases, Medical research, Oncology

## Abstract

The sub-study of the INSEMA trial (randomization-2) compares completion axillary lymph node dissection (cALND) with sentinel lymph node biopsy (SLNB) alone in cN0 patients with T1/T2 invasive breast cancer and one to three sentinel node macrometastases undergoing upfront breast-conserving surgery. The key secondary objective is to assess whether the SLNB-alone arm is non-inferior to cALND in terms of invasive disease-free survival (iDFS). Finally, 485 patients were recruited, and 386 patients (cALND: *N* = 169, SLNB alone: *N* = 217) were included in the per-protocol set. The median follow-up is 74.2 months. The 5-year iDFS analysis in the per-protocol set demonstrates a non-significant difference between study arms, with a hazard ratio (HR) of 1.69 (95% CI: 0.98-2.94) for SLNB alone compared to cALND. The 5-year iDFS rates are 86.6% (81.0%-90.7%) in the SLNB-alone arm and 93.8% (88.7%-96.6%) in the cALND arm (*P* = 0.058). The 5-year overall survival rates are 94.9% (90.6%-97.2%) in the SLNB-alone arm and 96.2% (91.7%-98.3%) in the cALND arm (*P* = 0.663). Locoregional recurrences (LRR) were infrequent, with 5-year incidence rates of 1.1% versus 0.0% (*P* = 0.405) in the SLNB-alone arm compared to cALND. In summary, no significant differences were observed between SLNB alone versus cALND for iDFS, overall survival, and LRR. Trial registration number: NCT02466737

## Introduction

The publication of the landmark American College of Surgeons Oncology Group (ACOSOG) Z0011 trial had a significant impact on the axillary management strategy for women with early breast cancer who did not receive neoadjuvant systemic therapy (NAST) and are pathologically sentinel lymph node-positive at diagnosis (after a clinically node-negative [cN0] presentation)^1^. The first results, after a median follow-up of 6.3 years, showed no difference between sentinel lymph node biopsy (SLNB) alone and completion axillary lymph node dissection (cALND) after breast-conserving surgery (BCS) with one or two metastases in the SLNB^2,3^. These data were confirmed by long-term analyses after a median follow-up of 9.3 years for overall survival (primary outcome), disease-free survival, and locoregional recurrences^4,5^.

Various limitations of the ACOSOG Z0011 trial, including the lack of preoperative axillary ultrasound (AUS), the significant enrollment of patients with nodal micrometastases, recruitment numbers lower than expected, and frequent protocol violations involving regional nodal irradiation (RNI)^6^, have led to the design of numerous validation trials. Four prospective randomized trials (SINODAR-ONE^7^, SENOMAC^8^, INSEMA (randomization-2)^9^, and POSNOC^10^) investigate the omission of cALND in sentinel lymph node-positive patients with upfront surgery. SINODAR-ONE, SENOMAC, and POSNOC recruited patients with one or two macrometastases in the sentinel nodes after BCS and mastectomy. The INSEMA sub-study enrolled only women with BCS. After protocol amendment #4 (September 2016), the maximum number of sentinel node macrometastases was increased to three.

Recently, 3-year survival and relapse rates after a median follow-up of 2.8 years for SINODAR-ONE patients demonstrated that the SLNB-alone arm was non-inferior to cALND^11^. The SENOMAC trialists’ group published secondary outcome data (5-year recurrence-free survival, RFS) after a median follow-up of 3.9 years, indicating that the omission of cALND was non-inferior to the more extensive surgery in patients also receiving nodal radiation therapy^12^. The primary end point of the POSNOC trial is the 5-year axillary recurrence rate; however, outcome data are not yet available.

We are now presenting the key secondary outcome data for the Intergroup-Sentinel-Mamma (INSEMA) trial. The goal of the INSEMA randomization-2 is to demonstrate that omission of cALND (SLNB alone) does not result in inferior invasive disease-free survival (iDFS) compared to the cALND arm in patients with one to three sentinel node macrometastases in early breast cancer treated with BCS and postoperative whole-breast irradiation (WBI).

## Results

### Patient characteristics

Patient and tumor characteristics for the per-protocol set are presented in Table 1. All baseline parameters were well-balanced between treatment arms except for the Ki-67 index. The percentage of tumors with a high proliferation rate (Ki-67 > 20%) was higher in the cALND arm compared to the SLNB alone arm (23.4 versus 15.4%; *P* = 0.06). The median age at diagnosis was 59.0 years (range 32.0–89.0; interquartile range [IQR]: 52.0–68.0). The median (IQR) preoperative tumor size, as determined by palpation, was 20 mm (15–20), and by imaging (95% based on sonography), 15 mm (11–19). Consequently, 81.1% of patients were diagnosed with a clinical T1 stage. In contrast to the preoperative evaluation, the rate of the pT2 stage increased to 39.4% after the final pathology report was documented. No difference was observed for medial tumor location between the study arms (cALND: 31.4% versus SLNB alone: 30.9%). Multifocal disease was more frequently diagnosed in the cALND arm (7.1 versus 3.2% in the SLNB alone arm; *P* = 0.081). The mean number of dissected sentinel lymph nodes was 2.4 (median 2.0; IQR: 1.0–3.0). The mean number of positive sentinel lymph nodes was 1.3 (median 1.0; IQR 1.0–1.0) without difference between study arms (*P* = 0.346). In the case of cALND, the mean number of dissected axillary lymph nodes was 13.2 (median 13.0; IQR: 10.0–15.0). The mean number of involved axillary lymph nodes in patients with cALND was 2.0 (median 1.0; IQR: 1.0–2.0). In the cALND arm, 19 patients (11.2%) had a pN2a stage.

**Table 1:** Patient and tumor characteristics (per-protocol set, *N* = 386)

Parameter	Category	cALND *N* (%) *N* = 169	SLNB alone *N* (%) *N* = 217	Overall *N* (%) *N* = 386
Age	<35 years	1 (0.6)	0 (0.0)	1 (0.3)
	35 to <50 years	29 (17.2)	34 (15.7)	63 (16.3)
	50 to <60 years	60 (35.5)	75 (34.6)	135 (35.0)
	60 to <70 years	47 (27.8)	57 (26.3)	104 (26.9)
	≥70 years	32 (18.9)	51 (23.5)	83 (21.5)
BMI	<30 kg/m^2^	117 (69.2)	164 (75.6)	281 (72.8)
	≥30 kg/m^2^	52 (30.8)	53 (24.4)	105 (27.2)
Preoperative tumor size^a^	≤2 cm	139 (82.2)	174 (80.2)	313 (81.1)
	>2 cm	30 (17.8)	43 (19.8)	73 (18.9)
Pathological tumor size	pTis	0 (0.0)	1 (0.5)	1 (0.3)
	pT1	99 (58.6)	129 (59.4)	228 (59.1)
	pT2	68 (40.2)	84 (38.7)	152 (39.4)
	pT3	2 (1.2)	3 (1.4)	5 (1.3)
Number of involved SLNs (only macrometastases)	1	127 (75.2)	171 (78.8)	298 (77.2)
	2	34 (20.1)	41 (18.9)	75 (19.4)
	3	8 (4.7)	5 (2.3)	13 (3.4)
Number of all involved LNs (SLNB + cALND)	1–3	150 (88.8)		
	4–9	19 (11.2)		
ER/PgR status	Negative	2 (1.2)	5 (2.3)	7 (1.8)
	Positive	167 (98.8)	212 (97.7)	379 (98.2)
HER2 status	Negative	162 (95.9)	205 (94.9)	367 (95.3)
	Positive	7 (4.1)	11 (5.1)	18 (4.7)
	Missing	0	1	1
Intrinsic subtype	HR+/HER2−	160 (94.7)	201 (93.1)	361 (93.8)
	TNBC	2 (1.2)	4 (1.9)	6 (1.6)
	HER2+	7 (4.1)	11 (5.1)	18 (4.7)
	Missing	0	1	1
Tumor grading	G1	45 (26.6)	63 (29.0)	108 (28.0)
	G2	115 (68.0)	143 (65.9)	258 (66.8)
	G3	9 (5.3)	11 (5.1)	20 (5.2)
Ki-67	≤20%	128 (76.6)	176 (84.6)	304 (81.1)
	>20%	39 (23.4)	32 (15.4)	71 (18.9)
	Missing	2	9	11
Histological subtype	Invasive carcinoma (NST)	125 (74.0)	155 (71.4)	280 (72.5)
	Invasive or mixed lobular carcinoma	22 (13.0)	25 (11.5)	47 (12.2)
	Other	22 (13.0)	37 (17.1)	59 (15.3)

*BMI* body mass index, *SLN* sentinel lymph node, *SLNB* sentinel lymph node biopsy, *LN* lymph node, *ER* estrogen receptor, *PgR* progesterone receptor, *NST* no special type, *cALND* completion axillary lymph node dissection.

^a^Based on the results of sonography. If those were missing, results from other assessments were used in the following order: mammography and magnetic resonance imaging.

### Postoperative radiotherapy

A total of 386 patients were treated with 3D-conformal radiotherapy (3D-CRT) using standard tangential fields (*N* = 216, 56.4%). The remaining patients (*N* = 132, 34.4%) received modern intensity-modulated radiotherapy (IMRT) techniques. The deep inspiration breath-hold technique was applied in 10 patients (2.6%). No differences were observed between randomized groups regarding the radiotherapy technique used.

Conventional fractionation was the preferred schedule for the application of WBI in the randomization-2 cases (*N* = 304, 80.4%), with significant differences between treatment arms (cALND: 87.0% versus SLNB alone: 75.1%; *P* = 0.004). Moderate hypofractionation was used for 74 patients (19.6%), with a higher frequency in patients undergoing SLNB alone (24.9%) compared to 13.0% in patients with cALND. Standard WBI values for planned target volume (PTV), dose median, and dose average were not different between study arms with respect to the fractionation schedule. A tumor bed boost was delivered to 325 patients (84.2%) with a higher application rate in the cALND cohort (88.8 versus 80.6% in the SLNB alone arm; *P* = 0.035). The boost timing was balanced (50.5% simultaneously versus 49.5% sequentially); 23 patients (7.2%) were treated with intraoperative boost irradiation.

Detected values for dose median and dose average for each axillary level are shown in Table 2. All dose parameters are presented as relative doses, expressed as a percentage of the prescribed breast dose, to avoid differences in absolute doses between conventionally fractionated and hypofractionated cases. Approximately 75% of patients included in the analysis of “dose median” in axillary level I (based on the first quartile value; Q1) were unintentionally treated with ≥80% of the prescribed median breast radiation dose (50.9 Gy with conventional fractionation; 40.7 Gy with moderate hypofractionation) without differences between treatment groups. Applied relative doses were lower in axillary levels II and III compared to level I values, with a non-significant trend for higher doses (levels II–III) in the cALND arm.

**Table 2:** Axillary dose parameters in INSEMA randomization-2 patients treated with postoperative whole-breast irradiation after breast-conserving surgery (per-protocol set)

Parameter		cALND *N* = 169	SLNB alone *N* = 217	Overall *N* = 386	*P* value
Axillary level I dose median (% of breast dose in Gy)	Mean	78.9	78.5	78.7	0.989
	SD	32.7	32.0	32.3	
	Median	94.6	95.0	94.8	
	Q1–Q3	83.5–97.8	77.9–98.0	79.6–98.0	
	Missing	44	45	89	
Axillary level I dose average (% of breast dose in Gy)	Mean	73.7	75.6	74.8	0.997
	SD	30.9	27.5	29.0	
	Median	88.5	84.9	86.8	
	Q1–Q3	68.0–95.0	65.7–95.2	65.7–95.2	
	Missing	36	40	76	
Axillary level II dose median (% of breast dose in Gy)	Mean	59.2	56.0	57.4	0.307
	SD	40.8	40.3	40.5	
	Median	81.7	74.6	77.2	
	Q1–Q3	10.1–97.3	8.7–95.6	9.4–96.4	
	Missing	43	45	88	
Axillary level II dose average (% of breast dose in Gy)	Mean	60.8	55.6	57.9	0.154
	SD	36.8	36.3	36.5	
	Median	72.0	68.6	69.6	
	Q1–Q3	27.0–96.6	16.9–91.6	18.7–95.0	
	Missing	35	41	76	
Axillary level III dose median (% of breast dose in Gy)	Mean	46.0	35.3	39.8	0.223
	SD	45.3	41.3	43.3	
	Median	11.7	9.3	10.0	
	Q1–Q3	3.6–97.6	3.2–87.9	3.4–95.2	
	Missing	43	45	88	
Axillary level III dose average (% of breast dose in Gy)	Mean	47.3	38.1	42.0	0.155
	SD	43.0	38.9	40.9	
	Median	26.6	22.1	23.0	
	Q1–Q3	6.0–96.4	4.1–85.2	4.8–93.8	
	Missing	36	41	77	

*Q1* first quartile, *Q3* third quartile, *SD* standard deviation, *SLNB* sentinel lymph node biopsy, *cALND* completion axillary lymph node dissection, *Gy* Gray.

The RNI was performed in 36.0% of patients with cALND, compared to 20.6% in the SLNB alone arm (*P* = 0.019). Predominantly, supraclavicular (34.8 versus 20.0%) and infraclavicular nodes (25.8 versus 14.3%) were included in the PTV, and less frequently, the internal mammary nodes (5.7 versus 0.8%). Notably, the RNI documentation started in March 2017 with the implementation of protocol amendment #4 (Table 3).

**Table 3:** Postoperative systemic therapy and regional nodal irradiation (RNI) in the per-protocol set (*N* = 386)

Parameter	Category	cALND *N*/*N* (%) *N* = 169	SLNB only *N*/*N* (%) *N* = 217	Overall *N*/*N* (%) *N* = 386	*P* value
Adjuvant chemotherapy	Yes	66/166 (39.8%)	73/217 (33.6%)	139/386 (36.3%)	0.239
	Missing	3	0	3	
Chemotherapy regimen	Anthracycline-based	2 (1.2)	5 (2.3)	7 (1.8)	
	Taxane-based	6 (3.6)	5 (2.3)	11 (2.9)	
	Anthracycline- and taxane-based	58 (34.9)	63 (29.0)	121 (31.6)	
	Missing	3	0	3	
Adjuvant endocrine therapy	Yes	156/165 (94.5%)	208/217 (95.9%)	364/382 (95.3%)	0.629
	Missing	4	0	4	
Other adjuvant therapy regimen	Anti-HER2 treatment	3 (1.8)	7 (3.3)	10 (2.6)	0.524
	Bisphosphonate	23 (13.9)	25 (11.6)	48 (12.6)	0.536
	Denosumab	1 (0.6)	2 (0.9)	3 (0.8)	1.0
RNI performed	Yes	32/89 (36.0%)	26/126 (20.6%)	58/215 (27.0%)	0.019
	Pts. before AM4	80	91	171	
RNI supraclavicular nodes	Yes	31/89 (34.8%)	25/125 (20.0%)	56/214 (26.2%)	0.018
RNI infraclavicular nodes	Yes	23/89 (25.8%)	18/126 (14.3%)	41/215 (19.1%)	0.036
RNI internal mammary nodes	Yes	5/88 (5.7%)	1/126 (0.8%)	6/214 (2.8%)	0.084

RNI documentation started in March 2017 after implementing protocol amendment #4 (AM4).

*Pts.* patients.

### Adjuvant systemic therapy

No differences in the application of postoperative systemic treatment were observed between randomization groups in the per-protocol set (Table 3). Slightly more patients received adjuvant chemotherapy in the cALND arm (39.8 versus 33.6% in the SLNB alone arm; *P* = 0.239). Identical chemotherapy application rates were observed among the intention-to-treat (ITT) population: 35.7% in the cALND arm versus 36.3% in the SLNB-alone arm (*P* = 0.924).

### End point analyses

**Fig. 1:**
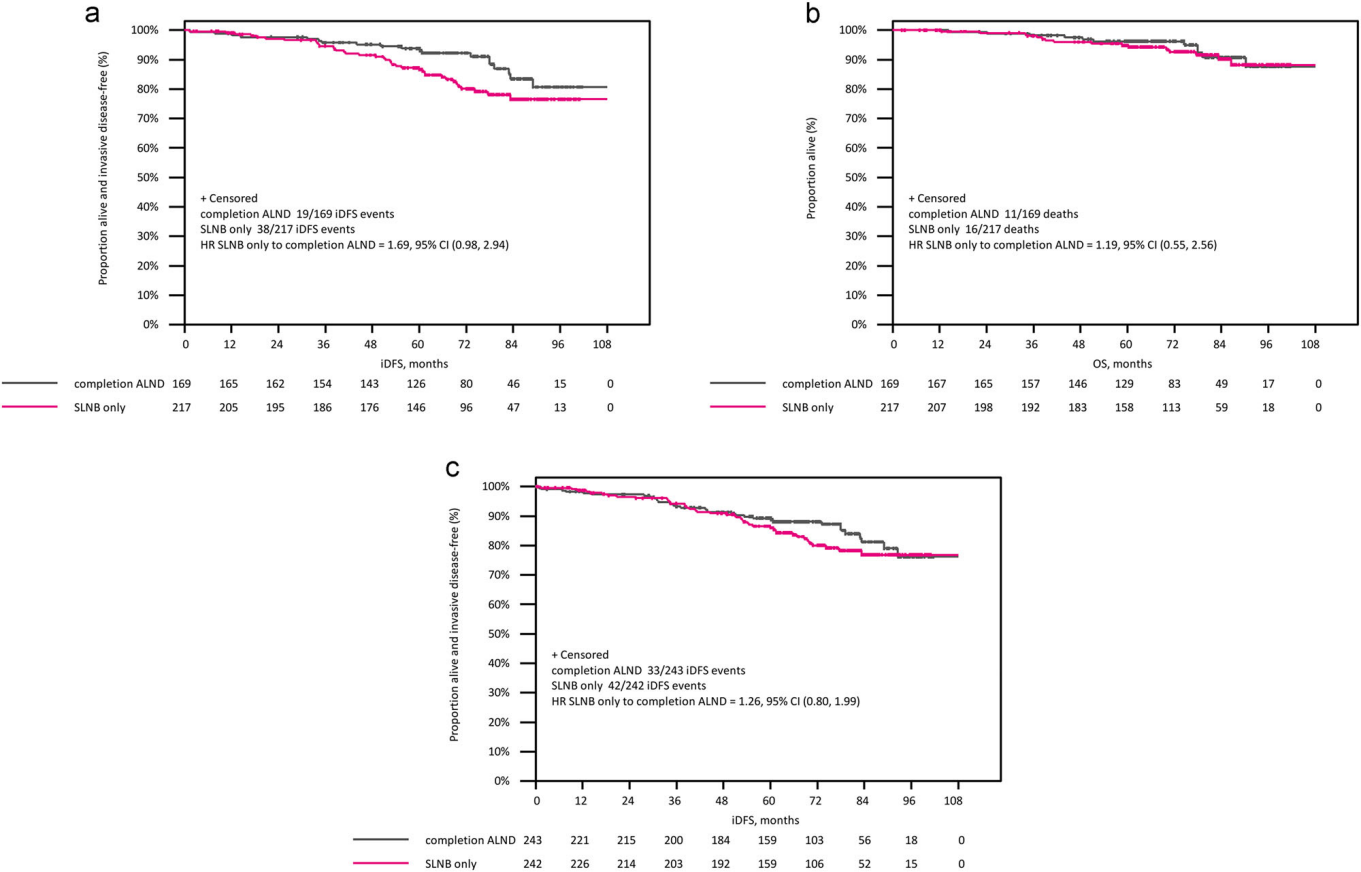
Secondary survival analyses for INSEMA randomization-2 population after a median follow-up of 74.2 months. Kaplan–Meier estimates of invasive disease-free survival (**a**) and overall survival (**b**) in the per-protocol set; Kaplan–Meier estimates for invasive disease-free survival in the intention-to-treat set (**c**).

The median follow-up is 74.2 months (6.2 years), with an overall follow-up completeness of 89.3%^13^. The iDFS analysis in the per-protocol set demonstrates a non-significant difference between study arms, with an HR of 1.69 (95% confidence interval [CI]: 0.98–2.94) for SLNB alone compared to cALND (Fig. 1a), indicating that non-inferiority could not be demonstrated. The estimated 5-year iDFS rates are 86.6% (81.0–90.7%) in the SLNB-alone arm and 93.8% (88.7–96.6%) in the cALND arm (log-rank *P* = 0.058).

The first iDFS events for SLNB alone versus cALND are listed in Table 4. Differences were observed when comparing distant relapse (SLNB alone: 6.9% versus cALND: 4.1%) and secondary malignancy rates (SLNB alone: 4.1% versus cALND: 2.4%). The estimated 5-year overall survival rates are 94.9% (90.6–97.2%) in the SLNB-alone arm and 96.2% (91.7–98.3%) in the cALND arm (Fig. 1b; log-rank *P* = 0.663).

**Table 4:** Summary of iDFS events in the per-protocol set

Parameter	Category	cALND *N* (%) *N* = 169	SLNB alone *N* (%) *N* = 217	Overall *N* (%) *N* = 386
iDFS event	No	150 (88.8)	179 (82.5)	329 (85.2)
	Yes	19 (11.2)	38 (17.5)	57 (14.8)
First iDFS event	Invasive locoregional relapse	2 (1.2)	4 (1.8)	6 (1.6)
	Invasive contralateral BC	0 (0.0)	1 (0.5)	1 (0.3)
	Distant relapse	7 (4.1)	15 (6.9)	22 (5.7)
	Secondary malignancy (not related to breast cancer)	4 (2.4)	9 (4.1)	13 (3.4)
	Death	6 (3.6)	9 (4.1)	15 (3.9)
Locoregional relapse	Axillary recurrence	0 (0.0)	1 (0.5)	1 (0.3)
	Invasive ipsilateral breast recurrence	2 (1.2)	3 (1.4)	5 (1.3)

*BC* breast cancer, *cALND* completion axillary lymph node dissection, *SLNB* sentinel lymph node biopsy, *iDFS* invasive disease-free survival.

Analyses among the sensitivity set (including patients without radiotherapy; *N* = 404) confirmed the results in the per-protocol set with estimated 5-year iDFS rates of 86.1% (80.5–90.2%) for the SLNB alone versus 93.3% (88.2–96.2%) for the cALND arm (log-rank *P* = 0.061). Among the ITT set, the iDFS analysis (Fig. 1c) shows no difference between study arms, with an HR of 1.26 (95% CI: 0.80–1.99) for SLNB alone compared to cALND. The distribution (SLNB alone versus cALND) of first iDFS events (*N* = 75) was slightly different from the per-protocol set: invasive locoregional recurrences (2.5 versus 1.6%), including axillary recurrences (0.8 versus 0.4%), invasive contralateral breast cancer (0.8 versus 0.0%), distant metastases (6.6 versus 4.5%), secondary malignancies (3.7 versus 2.9%), and deaths (3.7 versus 4.5%). The estimated 5-year iDFS rates (ITT set) are 86.0% (80.6–90.0%) in the SLNB-alone arm and 89.3% (84.3–92.8%) in the cALND arm (log-rank *P* = 0.314).

**Fig. 2:**
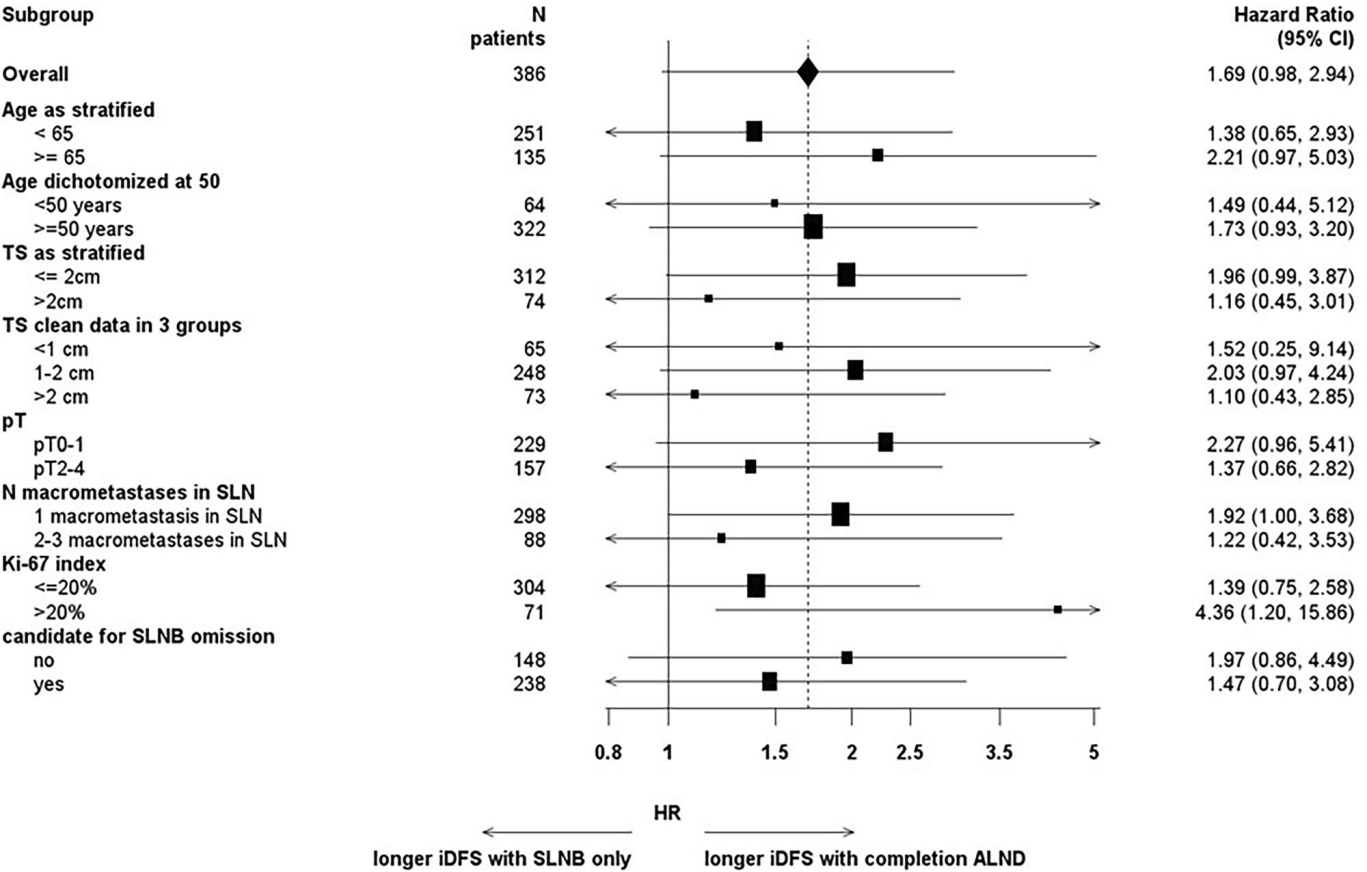
Univariate Cox regression forest plot for invasive disease-free survival in subgroups. ASCO guideline criteria for SLNB omission during breast-conserving therapy are: postmenopausal status (age ≥50 years), preoperative tumor size ≤2 cm, tumor grading G1-G2, and hormone receptor-positive/HER2-negative subtype. pT pathological tumor stage, *N* numbers, TS preoperative tumor size, based on sonography results. If those were missing, results from other assessments were used in the following order: mammography and magnetic resonance imaging.

Multivariate Cox regression analysis for iDFS, adjusted by stratification factors, showed that age ≥65 years and preoperative tumor size >2 cm, but not tumor grading G3, were related to worse iDFS (Table S1). The univariate Cox regression for iDFS in subgroups for age, tumor size, and number of macrometastases showed no substantial heterogeneity in HR (SLNB alone versus cALND) between subgroups (Fig. 2). Patients with highly proliferative tumors (Ki-67 > 20%) significantly benefited from the cALND regarding 5-year iDFS (HR = 4.36 [1.20–15.86] versus HR = 1.39 [0.75–2.58] for patients with a Ki-67 ≤ 20%). Patients potentially candidates for SLNB omission according to the published INSEMA randomization-1 results^14^ and updated ASCO guideline criteria^15^ had less benefit from the cALND in terms of 5-year iDFS compared to patients who are still candidates for the SLNB procedure (HR = 1.47 [0.70–3.08] versus HR = 1.97 [0.86–4.49]).

### Surgical complications

The rate of updated short-term surgery-related complications (Table S2) confirmed previously published data^16^. The long-term safety analysis demonstrates that patients in the SLNB alone arm benefited in terms of reduced lymphedema rate (6.6 versus 14.9%), arm/shoulder mobility restriction (2.5 versus 8.0%), and arm/shoulder movement pain (3.1 versus 5.8%), all parameters not resolved at the last follow-up visit (Table S3).

## Discussion

INSEMA randomization-2 provides additional data on the oncological safety of patients with cALND omission following a positive SLNB during BCS, with a strict indication for RNI and radiotherapy quality assurance. For the first time, these results suggest a potential impact of omission of the cALND on the 5-year iDFS. However, fair conclusion cannot be given due to the fact that the targeted number of patients (initially 1968) was not met. The objective of the randomization-2 was downgraded to a secondary outcome during recruitment period so that previous statistical assumptions for non-inferiority of the SLNB-only arm as co-primary end point are not appropriate for the current analysis.

The INSEMA randomization-2 is a validation trial with an identical follow-up (6.2 versus 6.3 years) to the first publication of the ACOSOG Z0011 data^2,3^. The number of recruited patients with macrometastases in the SLNB is comparable between the two studies; all patients underwent BCS in both trials. In contrast to Z0011 and other validation trials, women with three sentinel nodes macrometastases (3.4%) could be enrolled in the INSEMA sub-study.

Regarding overall survival, the primary end point of the Z0011 trial, our secondary outcome analyses confirm the previous data. No difference was observed between the treatment arms, with slightly higher estimated 5-year overall survival rates in the INSEMA randomization-2, indicating a different risk profile for the recruited patients. Rates for clinical T2 stage, hormone receptor negativity, and high tumor grading (G3) were higher among the Z0011 population^3^. Overall survival rates as a primary end point in other validation trials (SINODAR-ONE, SENOMAC) are only available for SINODAR-ONE (per-protocol set: *N* = 822). After a median follow-up of 34.0 months, the 5-year overall survival rates were 99.2% and 98.7% in the cALND and SLNB-alone arms, respectively^11^.

Similarly, the 5-year iDFS did not differ significantly between the two groups of axillary treatment among INSEMA randomization-2. However, the iDFS curves separate after a follow-up of 36 months, with numerically higher iDFS rates in the cALND arm. This iDFS curve splitting was more pronounced in the per-protocol and sensitivity cohorts compared to the ITT set. The reported absolute difference of 7.2% for the estimated 5-year iDFS rates (per-protocol set) is primarily attributed to higher rates of distant relapses, unrelated secondary malignancies, and deaths. However, based on the fact that there was only 1 axillary recurrence in the entire per-protocol population over 6 years, the benefit of cALND to prevent axillary recurrences is negligible, and it seems unlikely that omission of cALND in the setting of appropriate systemic therapy and unintentionally radiation coverage to the lower axilla in most cases translates to significantly worse distant events. In contrast, Z0011 and recently published validation trials reported no differences in secondary survival parameters comparing SLNB alone versus cALND; the 5-year disease-free survival rates were 83.9% and 82.2%, respectively, among the Z0011 population^3^. In the SINODAR-ONE per-protocol cohort, the 5-year RFS rates were 95.6% and 96.4%, respectively^11^. The per-protocol SENOMAC population comprised 2540 patients, with estimated 5-year RFS rates of 89.7% and 88.7%, respectively^12^.

The INSEMA cALND cohort is characterized by higher rates of postoperative chemotherapy, conventionally fractionated WBI, tumor bed boost application, and RNI. The imbalance in chemotherapy and RNI frequencies between the randomization-2 groups was expected, given the knowledge of the pN2a stage in the cALND arm (11.2%). However, this cannot fully explain the described effect on the 5-year iDFS among the INSEMA population. Higher rates of postoperative chemotherapy in the cALND arm were also reported for the SINODAR-ONE and SENOMAC patients with no impact on RFS^11,17^.

The de-escalation of axillary surgery during BCS must be discussed in the context of radiotherapy. According to the INSEMA protocol, the randomization-2 cohort is characterized by a representative proportion of patients treated with moderate hypofractionation (19.6%). The high rate of 84.2% for tumor bed boost application after BCS is comparable to the SINODAR-ONE trial data (68.8% with boost). Despite a higher use of RNI in the cALND arm, no differences between the arms of the INSEMA randomization-2 were observed regarding incidental doses to the axilla. Recently, the EBCTCG meta-analysis demonstrates that RNI reduces the rate of breast cancer recurrence and improves breast cancer-specific and overall survival after long-term follow-up^18^. The current ESTRO ACROP guideline recommends consideration of moderate hypofractionation for RNI^19^. The HypoG-01 and the DBCG Skagen-1 trials have established non-inferiority of moderate hypofractionation for lymphedema. However, conflicting results have been reported regarding oncological outcomes. The HypoG-01 trialists demonstrated significant improvements in locoregional recurrence-free survival, disease-free survival, and overall survival with moderate hypofractionation^20^. In contrast, the DBCG Skagen trial-1 found that breast cancer-specific mortality was higher with moderate hypofractionation^21^. Again, while differences in RNI use and fractionation regimens may have affected the outcome in INSEMA randomization-2, the minor differences in treatment patterns do not fully explain the observed differences in the 5-year iDFS.

The consequences of omitting cALND after one to three macrometastases in the SLNB (including the questionable trend for impaired 5-year iDFS and lack of information for postoperative treatment indications, such as RNI and chemotherapy) must be weighted against the benefits of improved quality-of-life (QoL) and reduced risk of long-term complications. The large number of patients excluded from the per-protocol analysis (*N* = 66) due to not accepting randomization to the cALND arm suggests that both patients and providers are choosing to avoid cALND over the timeframe of the INSEMA study.

This INSEMA Rando2 analysis has numerous strengths. First, data are based on the longest median follow-up and the highest axillary tumor burden of all Z0011 validation trials. Second, patient-reported outcomes and prospective assessment regarding incidental axillary irradiation dose values during WBI are secondary outcomes for the study population. Third, target volumes for postoperative radiotherapy were predefined, thereby avoiding potential overtreatment.

In addition, our study has several limitations. First, the target recruitment number for the second randomization was not achieved, leading to an underpowered sub-study with only 20–25% of the planned enrollment. Second, major crossover between per-protocol and ITT cohorts lead to incongruent results comparing 5-year iDFS rates. Finally, the median follow-up of 6.2 years is appropriate for reporting 5-year survival data, but it may miss late recurrences in hormone receptor-positive diseases^22^. The analysis of 10-year survival data is planned for September 2029.

In conclusion, INSEMA randomization-2 demonstrated no significant differences between cALND versus SLNB alone in sentinel node-positive patients with early breast cancer and primary breast-conserving therapy. From a statistical point of view, our results regarding observed 5-year iDFS differences may be inconclusive and do not necessarily represent a failure to demonstrate non-inferiority of the SLNB-only arm. In the setting of increased use of RNI for any macrometastatic node-positive disease in the radiation oncology world, our results do not support the indication for cALND in patients with one to three sentinel node macrometastases.

## Methods

A comprehensive review of the INSEMA trial design was published in December 2024, together with the report of primary outcome data of the INSEMA randomization-1. Patients were first randomized to either no axillary surgery or SLNB in a 1:4 allocation. The complete omission of surgical axillary staging was non-inferior to SLNB, analyzing the 5-year iDFS after a median follow-up of 6.1 years^14^. The Clinical Trial Number is NCT02466737. The date on which the study record was first available on ClinicalTrials.gov was 2015-JUN-09.

### Patients

Women with breast cancer up to 5 cm (cT1/T2 stages), cN0 status (clinically and per imaging [iN0]), and upfront BCS were eligible. Patients with SLNB and pN+(sn) status (1–3 macrometastases) were randomized (1:1 ratio) to either SLNB alone or cALND. During follow-up, patients were assessed according to standard clinical practice.

**Fig. 3:**
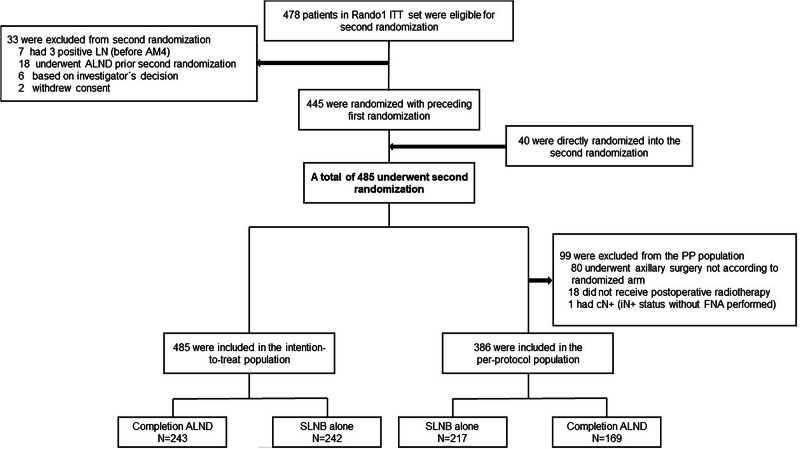
Flow diagram for randomized patients and the analysis populations for INSEMA randomization-2. SLNB sentinel lymph node biopsy, ITT intention-to-treat, PP per-protocol, SLN sentinel lymph node, ALND axillary lymph node dissection, LN lymph node, AM4 protocol amendment #4, cN+ clinically node-positive, iN+ node-positive per imaging, FNA fine-needle aspiration.

Four hundred eighty-five patients were recruited for the second randomization (ITT set: *N* = 243 with cALND versus *N* = 242 with SLNB alone). After excluding 99 patients, 386 patients (cALND: *N* = 169, SLNB alone: *N* = 217) were included in the per-protocol set (Fig. 3). The main reasons for excluding patients (20.4%) were axillary surgery not according to the randomized arm (cALND: *N* = 66 versus SLNB alone: *N* = 14) and no application of postoperative radiotherapy (cALND: *N* = 7 versus SLNB alone: *N* = 11). The safety set was used to analyze both short-term and long-term surgical complications.

### Local treatment

The preoperative diagnostic workup included a routine AUS performed before biopsy. All patients underwent ipsilateral BCS with postoperative WBI regardless of the intrinsic subtype. Conventional fractionation or moderate hypofractionation was an option using 3D-CRT or IMRT techniques for WBI. The axilla was not explicitly targeted. RNI was only recommended for patients with four or more positive axillary lymph node metastases as reported in the final pathology. Boost irradiation of the tumor bed was generally recommended but could be omitted in selected patients (those aged >60 years, with small tumor sizes, and favorable prognostic factors). The use of partial breast irradiation alone was not allowed. The first three radiotherapy plans treated at each center underwent central quality assurance^23^. Dosimetric data for axillary lymph node levels I-III were prospectively collected for the entire study population.

### Trial end points

The iDFS, as the primary outcome for the first randomization, is defined as the period between randomization and the first event (locoregional or distant invasive recurrence, death from any cause, contralateral invasive breast cancer, or second primary invasive cancer [non-breast])^24^. Due to the number of SLNB-positive patients being fewer than expected, the iDFS analysis for the second randomization was downgraded from a co-primary to a key secondary outcome following protocol amendment #5 (December 2018). Other secondary end points were overall survival, locoregional disease-free survival, ipsilateral axillary recurrence rate, distant disease-free survival, QoL analyses, and dose distribution in ipsilateral axilla levels I-III during radiotherapy.

### Statistical analysis

The following assumptions were made for the second randomization: the 5-year iDFS for optimally treated patients with one to three sentinel node macrometastases was considered 81%, and the non-inferiority margin was defined as the SLNB-alone group having a 5-year iDFS of not less than 76.5% (upper 95% CI end for HR < 1.271). The overall error rate of a false-positive outcome (α) is 5%, and the adjusted α for the second randomization was 3.61%. The error rate for a false-negative result (β) was set to 20%, i.e., the power of the trial was set to 80% for the difference in clinical interest. The calculated number of patients included in the per-protocol set for the second randomization was initially 1968. An event-driven final efficacy analysis was planned when 484 events occurred.

However, this target number of randomization-2 was not achieved for various reasons. First, the observed sentinel node-positivity rate for one to three macrometastases was lower than expected, at only 11.3% among the first INSEMA randomization cohort. Second, the recruitment rate directly for randomization-2 was below the planned numbers. Third, the ACOSOG Z0011 publication of long-term follow-up data in 2016/2017 led to modifications in the German guidelines and a decrease in the acceptance of the INSEMA randomization-2 design among physicians and patients. The INSEMA protocol amendment #5, which includes downgrading the iDFS analysis of randomization-2 to a secondary outcome without statistical assumptions, was released following a recommendation from the Independent Data Monitoring Committee.

Due to the non-inferiority study design, the key secondary end point analysis was performed on the per-protocol set^25,26^. All outcome results will be reported for both the per-protocol and ITT sets, as well as in a sensitivity analysis that includes patients who did not receive radiotherapy.

The analyses were performed using data available as of August 30, 2024, following 5.3 years of follow-up since the last patient was enrolled. The Kaplan–Meier product-limit method estimated 5-year iDFS rates (reported with the two-sided 95% CI). The non-inferiority was tested based on the 95% CI of the HR from the Cox proportional hazard model to exclude the HR of 1.271, but with no conclusive value for the key secondary objective. A multivariate Cox proportional hazards model adjusted HRs for stratification factors (age, tumor size, and tumor grading). The homogeneity of findings was explored in subgroups defined by age, tumor size, the number of macrometastases, and Ki-67 index using univariate Cox regression analyses. The Pocock minimization method^27^ was used for treatment allocation, stratified according to defined stratification criteria.

Analyses were performed using SAS® (Statistical Analysis Software; Cary, NC, USA) version 9.4 with SAS Enterprise Guide 8.3. There was no prespecified plan to adjust for multiple comparisons. All CIs and tests were two-sided; the widths of the CIs are not adjusted for multiple comparisons and should not be used in place of hypothesis testing of secondary outcomes.

### Ethics approval declaration

All patients provided informed written consent. The trial was conducted and monitored according to Good Clinical Practice guidelines based on the Declaration of Helsinki. After approval by the local independent review board at the University of Rostock (Germany, registration number HV-2011-0010), INSEMA randomization-2 enrolled women aged 18 years or older between September 2015 and April 2019 at 114 German and six Austrian study sites.

## Supplementary information

Supplementary Tables

Supplementary Information

## Data Availability

All available data are presented in the current manuscript.
