# Supplementary material for: Axillary surgery in patients with sentinel node macrometastases: secondary results of the randomized INSEMA trial
Source: NPJ Breast Cancer. 2026 Jan 27;12:19. doi: 10.1038/s41523-026-00902-7 (PMC12855813; doi:10.1038/s41523-026-00902-7)
Supplement: Supplementary file 1 — Supplementary Tables [file 41523_2026_902_MOESM1_ESM.pdf]

Table S1: Multivariate Cox regression adjusted by stratification factors for invasive disease-free survival in the per-protocol set (patients N=386; events N=57).

* Based on results of sonography. If those were missing, results from other assessments were used in the following order: mammography, magnetic resonance imaging.

Parameter	Value	HR for multivariate Cox regression	95%CI for HR
Study arm	cALND<br>SLNB alone	-<br>1.689	 (0.973-2.933)
Age (years)	<65<br>≥65	-<br>1.694	 (1.007-2.85)
Preoperative tumor size*	≤2 cm<br>>2 cm	-<br>2.036	 (1.154-3.591)
Tumor grading	G1/G2<br>G3	-<br>1.337	 (0.477-3.752)

Table S2: Clinician-reported surgical complications among INSEMA randomization-2 (cALND versus SLNB alone) observed within four weeks postoperatively (safety set; patients who received axillary surgery outside of the randomized allocation were analyzed as treated).

Parameter	Parameter value	cALND N/N (%) (N=191)	SLNB alone N/N (%) (N=294)	Overall N/N (%) (N=485)	P-value
Any surgical complication	yes	66 / 188 (35.1%)	60 / 286 (21.0%)	126 / 474 (26.6%)	<0.001
	missing	3	8	11	
Damage of vessels	yes	1 / 188 (0.5%)	0 / 286 (0.0%)	1 / 474 (0.2%)	0.397
	missing	3	8	11	
Damage of nerves	yes	2 / 188 (1.1%)	1 / 286 (0.3%)	3 / 474 (0.6%)	0.566
	missing	3	8	11	
Seromas in the breast and/or axilla	yes	23 / 188 (12.2%)	22 / 286 (7.7%)	45 / 474 (9.5%)	0.110
	missing	3	8	11	
Haematomas	yes	13 / 188 (6.9%)	24 / 286 (8.4%)	37 / 474 (7.8%)	0.604

Parameter	Parameter value	cALND N/N (%) (N=191)	SLNB alone N/N (%) (N=294)	Overall N/N (%) (N=485)	P-value
	missing	3	8	11	
Lymphedema	yes	8 / 188 (4.3%)	1 / 286 (0.3%)	9 / 474 (1.9%)	0.003
	missing	3	8	11	
Wound infections	yes	10 / 188 (5.3%)	5 / 286 (1.7%)	15 / 474 (3.2%)	0.057
	missing	3	8	11	
Axillary web syndrome (cording)	yes	2 / 188 (1.1%)	1 / 286 (0.3%)	3 / 474 (0.6%)	0.566
	missing	3	8	11	
Arm or shoulder mobility restriction	yes	19 / 188 (10.1%)	14 / 286 (4.9%)	33 / 474 (7.0%)	0.041
	missing	3	8	11	
Arm or shoulder mobility pain	yes	20 / 188 (10.6%)	14 / 286 (4.9%)	34 / 474 (7.2%)	0.028
	missing	3	8	11	
Weakness	yes	4 / 188 (2.1%)	2 / 286 (0.7%)	6 / 474 (1.3%)	0.220

Parameter	Parameter value	cALND N/N (%) (N=191)	SLNB alone N/N (%) (N=294)	Overall N/N (%) (N=485)	P-value
	missing	3	8	11	
Pulmonary embolism	yes	0 / 188 (0.0%)	0 / 286 (0.0%)	0 / 474 (0.0%)	n.a.
	missing	3	8	11	
Thrombosis	yes	1 / 188 (0.5%)	0 / 286 (0.0%)	1 / 474 (0.2%)	0.397
	missing	3	8	11	
Brachial plexus injury	yes	0 / 188 (0.0%)	0 / 286 (0.0%)	0 / 474 (0.0%)	n.a.
	missing	3	8	11	
Paresthesias	yes	11 / 188 (4.8%)	5 / 286 (1.7%)	16 / 474 (3.4%)	0.019
	missing	3	8	11	
Other	yes	8 / 188 (4.3%)	8 / 285 (2.8%)	16 / 473 (3.4%)	0.441
	missing	3	9	12	

Parameter	Parameter value	cALND N/N (%) (N=191)	SLNB alone N/N (%) (N=294)	Overall N/N (%) (N=485)	P-value
Not specified	yes	0 / 189 (0.0%)	1 / 286 (0.3%)	1 / 475 (0.2%)	1.000
	missing	2	8	10	

Table S3: Long-term follow-up (FU) of key parameters for clinician-reported surgical complications among INSEMA randomization-2 (cALND versus SLNB alone) observed within 60 months postoperatively (safety set; patients who received axillary surgery outside of the randomized allocation were analyzed as treated).

Parameter	Parameter value	cALND N/N (%) N=191	SLNB alone N/N (%) N=294	Overall N/N (%) N=485	P-value
Lymphedema	yes	79 / 188 (42.0%)	75 / 287 (26.1%)	154 / 475 (32.4%)	<0.001
	resolved at last FU	51 / 188 (27.1%)	56 / 287 (19.5%)	107 / 475 (22.5%)	
	missing	3	7	10	
Arm or shoulder mobility restriction	yes	66 / 188 (35.1%)	59 / 287 (20.6%)	125 / 475 (26.3%)	<0.001
	resolved at last FU	51 / 188 (27.1%)	52 / 287 (18.1%)	103 / 475 (21.7%)	
	missing	3	7	10	
Arm or shoulder mobility pain	yes	64 / 188 (34.0%)	68 / 287 (23.7%)	132 / 475 (27.8%)	0.016
	resolved at last FU	53 / 188 (28.2%)	59 / 287 (20.6%)	112 / 475 (23.6%)	

Parameter	Parameter value	cALND<br>N/N (%)<br>N=191	SLNB alone<br>N/N (%)<br>N=294	Overall<br>N/N (%)<br>N=485	P-value
	missing	3	7	10	
